# App use, physical activity and healthy lifestyle: a cross sectional study

**DOI:** 10.1186/s12889-015-2165-8

**Published:** 2015-08-28

**Authors:** Joan Martine Dallinga, Matthijs Mennes, Laurence Alpay, Harmen Bijwaard, Marije Baart de la Faille-Deutekom

**Affiliations:** School of Sports and Nutrition, Amsterdam University of Applied Sciences, Dr. Meurerlaan 8, 1067 SM Amsterdam, The Netherlands; Faculty of Health, Sports and Social Work, Inholland University of Applied Sciences, Blijdorplaan 15, 2015 CE Haarlem, The Netherlands

## Abstract

**Background:**

Physical inactivity is a growing public health concern. Use of mobile applications (apps) may be a powerful tool to encourage physical activity and a healthy lifestyle. For instance, apps may be used in the preparation of a running event. However, there is little evidence for the relationship between app use and change in physical activity and health in recreational runners. The aim of this study was to determine the relationship between the use of apps and changes in physical activity, health and lifestyle behaviour, and self-image of short and long distance runners.

**Methods:**

A cross sectional study was designed. A random selection of 15,000 runners (of 54,000 participants) of a 16 and 6.4 km recreational run (Dam tot Damloop) in the Netherlands was invited to participate in an online survey two days after the run. Anthropometrics, app use, activity level, preparation for running event, running physical activity (RPA), health and lifestyle, and self-image were addressed. A chi-squared test was conducted to analyse differences between app users and non-app users in baseline characteristics as well as in RPA, healthy lifestyle and perceived health. In addition, a multivariate logistic regression analysis was performed to determine if app use could predict RPA, perceived health and lifestyle, and self-image.

**Results:**

Of the 15,000 invited runners, 28 % responded. For both distances, app use was positively related to RPA and feeling healthier (*p* < 0.05). Also, app use was positively related to feeling better about themselves, feeling like an athlete, motivating others to participate in running, and losing weight (*p* < 0.01). Furthermore, for 16 km runners app use was positively related to eating healthier, feeling more energetic and reporting a higher chance to maintain sport behaviour (*p* < 0.05).

**Conclusions:**

These results suggest that use of mobile apps has a beneficial role in the preparation of a running event, as it promotes health and physical activity. Further research is now needed to determine a causal relationship between app use and physical and health related behaviour.

**Electronic supplementary material:**

The online version of this article (doi:10.1186/s12889-015-2165-8) contains supplementary material, which is available to authorized users.

## Background

Benefits of physical activity have often been studied and include improved health and reduced mortality rates [1–3]. However, actually becoming physically active is a challenge for many. In the Netherlands research shows that 41 % percent of all adults do not comply with the Dutch Public Health Physical Activity Guideline (at least 30 min of moderate to vigorous physical activity during at least 5 days of the week) [4]. Moreover, only 20 % of Dutch adults meet the Strenuous Intensity Physical Activity Guideline of at least three times a week 20 min of vigorous exercise [4]. Physical inactivity is a growing public health concern in the Netherlands as well as in other Western countries. Significant health problems such as increased morbidity and mortality attributable to cardiovascular disease, diabetes, cancers and increased risk of depression may arise if the amount of physical activity in the general population does not increase [5–9].

There is need for innovative ways to promote physical activity and a healthy lifestyle. One promising development is the use of smartphones during exercise. Use of mobile applications (apps) may be a powerful tool to encourage physical activity and health [10, 11]. Apps are accessible, have a large reach, and have multiple functionalities, such as interactive possibilities and feedback opportunities [12, 13]. Although more than 17,000 health and fitness apps have been developed and are available for the public [12], the literature considering the relationship of app use and health and physical activity is scarce.

However, preliminary evidence is promising [11, 14, 15]. Two reviews and one meta-analysis demonstrated positive effects of mobile phone interventions, interventions with mobile technology, and interventions with remote and web interventions in healthy, inactive and overweight individuals [11, 14, 15]. The mobile phone interventions were often combined with additional education, self-reporting of frequency and type of use of the program or telephone calls. The positive effects of these interventions included increased physical activity (expressed by total time, number of occasions of physical activity and energy expenditure), cardiovascular fitness and reduced overweight [11, 14, 15]. Small to moderate effect sizes were reported [14, 15]. Nevertheless, in these three reviews few interventions were included that used apps. Moreover, in some studies additional interventions were provided next to the mobile phone and app interventions, therefore based on those studies no conclusions can be drawn regarding to the isolated effects of apps on physical activity. Another recent review demonstrated modest effects of app based interventions on physical activity expressed by step count [16]. It should be noted that the apps were often combined with external pedometers, small sample sizes were included, small increases in step counts and a short duration of interventions was presented [16]. However, a recent study has shown promising results of the isolated effect of app use [17]. This study demonstrated that use of a Web-based app on lifestyle indicators decreased weight and increased physical activity of people [17]. Moreover, app users presented a higher chance to maintain a healthy lifestyle [17]. In summary, few studies have examined the effect of app use on changes in physical activity and health.

In recreational running the use of apps is high and emerging and several apps have been developed to assist individuals in their running exercise. Previous research has shown that recreational running or participation in a running mass event could also be a potential health and physical activity promoting activity [18–20]; Chatton and Kayser showed that participants in a 16 km run were more active than the general population and better in shape [18]. Additionally, in the preparation for a 5 and a 10 km run participants increased physical activity [19, 20]. A majority of participants train in preparation for running events; some of them exercise individually and some of them in a running group [21, 22]. Potentially, app use could assist runners to increase motivation, to increase activity level and set goals during the preparation for a running event. Perhaps the use of apps could assist runners to increase running physical activity and to live and feel healthier. Therefore, the aim of this study was to determine the relationship between the use of apps and changes in physical activity and health and lifestyle behaviour of short and long distance runners. More specific, we were interested in training volume, alcohol intake, smoking behaviour, and lifestyle (e.g. weight loss and eating behaviour).

## Methods

### Study design and participants

A cross sectional study was designed to analyse the relationship between app use and physical activity, health and lifestyle of recreational runners. On September 21^st^ 2014 the 30th Dam tot Damloop, a running event, was organized in Amsterdam, the Netherlands. The organization of the running event randomly selected and invited 15,000 runners out of 54,410 participants (16 and 6.4 km) to participate in an online survey. Runners of all levels were invited to participate. Participation in the run was either on an individual basis, with a company or for a charity. Inclusion criteria were (a) ≥18 years and (b) signed informed consent. Exclusion criteria were (a) participating in both distances or (b) leaving all questions unanswered after informed consent.

Two days after participation to the event, an email invitation including a link to the online survey was sent to the random selection of participants. After one week, a reminder was sent to the participants who had not responded yet. This online survey was based on a previously developed survey [23], with additional items for this specific running event. An additional file presents the survey questions (see Additional file 1). In the introduction of the survey the purpose of the study was explained and confidentiality was guaranteed. Furthermore, it was ascertained that participation was voluntary and that the participant was allowed to quit at any time. Responding to the questionnaire took approximately 15 min. The ethical approval was not required in the Netherlands, however the research was conducted in line with the Helsinki Declaration.

### Key measures

#### Dependent variables

Running physical activity (RPA) was collected. Participants were invited to report on two occasions (before their training phase (baseline) and during training phase) how many kilometres per week they ran (<5 km a week, between 5 and 10, between 10 and 20 a week, between 20 and 30 a week and more than 30 km per week). In addition, the survey included questions regarding health and lifestyle. Alcohol consumption (glasses per week) and frequency of smoking (per day) was asked before their training phase and during training phase. Additionally, participants were requested to indicate whether participation in the run affected their health (no effect, feel much healthier, feel healthier, feel less healthy, feel much less healthy). Moreover, participants indicated if the run influenced their body weight, diet, and energy level (totally agree, agree, neutral, disagree, totally disagree). To gain insight in self-image, participants were asked whether the run influenced their perception concerning a healthy lifestyle (totally agree, agree, neutral, disagree, totally disagree). Items included were: performing sports is good for me, chance of maintaining physical activity, feeling better about oneself, no change in lifestyle, and feeling tired more often.

#### Potential prediction variables

Participants indicated if they used an app or other training tool. Additionally, we collected information about several variables that needed to be controlled for (gender, age, body mass index (BMI)). To calculate age, date of birth was asked. Subsequently, age was calculated by subtracting the year of birth from 2014. BMI (kg/m^2^) was used as a proxy of body composition and calculated as self-reported body weight (kg) divided by the square of height (m). We used the WHO categories for the classification: BMI < 18.5 means underweight, [18.5, 25) equals normal weight, [25, 30) means overweight and ≥ 30 corresponds to obese [24]. In addition, information was collected to determine the participant’s preparation for the event, fitness state, and experience with running/sports. As an indication, exercise frequency, in number of training sessions per year, was requested [25]. Participants were asked whether they had participated before in this running event (and if so the number of previous participations) to estimate experience with running events. The training period that participants scheduled to prepare for this running event was asked as well. Participants could choose between categories: no training/barely, 1–5 weeks, 6–11 weeks, 12 weeks or more, no specific training/train all year and don’t know/no answer. Participants indicated self-reported finishing time in hours and minutes as well.

### Data reduction

The difference in RPA between baseline and training phase was calculated. For all participants it was assessed whether the RPA was increased or not. Furthermore, the difference in consumption of alcohol and smoking between baseline and training phase was calculated. Calculations were performed to examine if these two factors were decreased or not. For the outcome of perceived health it was determined whether participants felt healthier or not. Answers on theses concerning healthy lifestyle and self-image were reduced from five to two categories; we calculated if the participants agreed or answered neutral/disagreed with the theses about these topics.

### Statistical analysis

SPSS version 20.0 was used for all calculations. For both distances, means and standard deviations (SD’s) were calculated for age, BMI and exercise frequency. The data was checked for outliers. For the categorical variables, frequency and percentage were calculated.

We used a chi-squared test to determine differences between app users and non-app users in baseline characteristics as well as in physical activity, healthy lifestyle and perceived health during training phase. In addition, a multivariate logistic regression analysis was performed to determine if app use could predict changes in RPA, health and lifestyle, and self-image. Outcome variables were effects on RPA (increased, not increased), health (healthier/not healthier), alcohol consumption (more/not more), smoking (more/not more), eat healthier (agree/disagree), energy level (agree/disagree), performing sports is good for me (agree/disagree), chance of maintaining physical activity (agree/disagree), feeling better about oneself (agree/disagree), no change in lifestyle (agree/disagree), lose weight (agree/disagree), and feel tired more often (agree/disagree). In these logistic regression analyses, we controlled for age, gender, BMI, kilometres per week before preparation and exercise frequency in last year. Separate analyses were performed for the 16 and the 6.4 km. The alpha level was set at α ≤ 0.05 *a priori*.

## Results

**Fig. 1:**
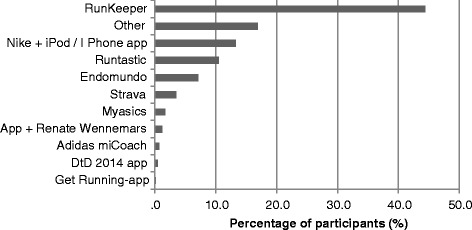
Apps used in preparation for the 16 and 6.4 km recreational run

Of all invited runners 4307 (28 %) agreed to participate in the survey, of which 2838 runners participated in the 16 km and 1341 in the 6.4 km. Table 1 presents the subject characteristics of male and female 16 and 6.4 km runners. Hundred-twelve participants participated in both distances and 507 participants reported too much missing values and were therefore excluded. The type of apps used by participants is shown in Fig. 1. Most participants used Runkeeper (44.4 %) in their preparation. The category ‘other apps” was the second largest app type chosen by participants (16.9 %), these were the apps that were not mentioned in the answer options.

**Table 1:** Subject characteristics of 16 and 6.4 km runners

		16 km		6.4 km	
		Males	Females	Males	Females
Variable		M ± SD	M ± SD	M ± SD	M ± SD
Age (years)		42.19 ± 10.73	37.11 ± 10.26	42.01 ± 11.39	36.33 ± 10.31
Training sessions per year (n/year)		120.91 ± 56.81	121.31 ± 55.39	101.17 ± 57.90	99.97 ± 56.08
		N (%)^a^	N (%)^a^	N (%)^a^	N (%)^a^
BMI category	Underweight	20 (1.0)	46 (2.2)	5 (0.5)	40 (4.1)
	Normal weight	756 (36.1)	646 (30.9)	97 (10.0)	443 (45.5)
	Overweight	481 (23.0)	143 (6.8)	140 (14.4)	249 (25.6)
Use of app during training	Yes	736 (28.5)	543 (21.0)	160 (13.5)	537 (45.3)
	No	830 (32.1)	477 (18.4)	140 (11.8)	349 (29.4)
Duration training period	No training/ barely	114 (4.4)	38 (1.5)	37 (3.1)	97 (8.2)
	1–5 weeks	129 (5.0)	88 (3.4)	46 (3.9)	100 (8.4)
	6–11 weeks	183 (7.1)	125 (4.8)	33 (2.8)	117 (9.9)
	12 weeks or more	225 (8.7)	218 (8.4)	38 (3.9)	129 (10.9)
	No separate training period	909 (35.2)	546 (21.1)	142 (12.0)	433 (36.5)
	Don’t know/no answer	6 (0.2)	3 (0.1)	5 (0.4)	9 (0.8)
Kilometres before	< 5 km/week	229 (9.1)	134 (5.3)	86 (7.6)	328 (28.9)
	5–10 km/week	318 (12.6)	332 (13.2)	96 (8.5)	314 (27.7)
	10–20 km/week	473 (18.8)	307 (12.2)	68 (6.0)	165 (14.5)
	20–30 km/week	301 (11.9)	162 (6.4)	25 (2.2)	34 (3.0)
	> 30 km/week	202 (8.0)	64 (2.5)	12 (1.1)	7 (0.6)

^a^Total N varies due to missing values

### Differences app and non-app users

#### Baseline characteristics

A significant association was found between app use and gender for both distances. More app users were female (16 km: Chi-squared = 4.90, *p* = 0.027; 6.4 km: Chi-squared = 9.61, *p* = 0.002). In addition, app users were significantly younger compared to non-app users (16 km: t = -12.09, df = 2456.56, *p* < 0.001; 6.4 km: t = −4.24, df = 879.92, *p* < 0.001) and trained less often in a year (16 km: t = −5.58, df = 2542.24, *p* < 0.001; 6.4 km: t = −2.44, df = 969.84, *p* = 0.015). In the 6.4 km runners, app use was associated with BMI category (Chi-squared = 7.45, *p* = 0.024); app users were more often overweight. We found a significant association between app use and kilometres per week that participants ran before the preparation phase (16 km: Chi-squared = 87.48, *p* < 0.001; 6.4 km: Chi-squared = 16.10, *p* = 0.003). In general, it seemed that app users trained fewer kilometres before they had started the preparation for the running event, compared to non-app users. A significant association between app use and duration of training period was found as well (16 km: Chi-squared = 69.36, *p* < 0.001; 6.4 km: Chi-squared = 30.16, *p* < 0.001). For the 16 km, there were more app users who trained 12 weeks or more and who did not schedule a specific training period for this event compared to the non-app users. For the 6.4 km, app users trained more often 6 to 11 weeks and 12 weeks or more compared to non-app users, whereas non-app users more often did not train or trained barely compared to app users.

#### Outcome variables

Table 2 shows the differences between app users and non-app users in RPA, perceived health and lifestyle, and self-image. App users increased more often their RPA, felt healthier, ate healthier (6.4 km no significant difference), felt more energetic, felt that they had a higher chance of maintaining sport behaviour, felt better about themselves, felt more like an athlete, changed their lifestyle, stimulated others to perform sport and lost weight.

**Table 2:** Differences between app users and non-app users in RPA, perceived health and lifestyle, and self-image

		16 km				6.4 km			
		App use	No app use	Chi^2^	P	App use	No app use	Chi^2^	P
		N (%)	N (%)			N (%)	N (%)		
RPA	Decreased/same	624 (23.7)	821 (31.1)	55.49	< 0.001	467 (39.1)	369 (30.9)	17.22	< 0.001
	Increased	689 (26.1)	504 (19.1)			246 (20.6)	112 (9.4)		
Perceived health	Not healthier	497 (18.2)	722 (27.5)	72.71	< 0.001	294 (23.5)	268 (21.4)	18.36	< 0.001
	Healthier	863 (31.6)	646 (23.7)			443 (35.4)	246 (19.7)		
Smoking behaviour^a^	More/equal	164 (43.3)	111 (29.3)	0.11	0.814	91 (52.3)	52 (29.9)	2.16	0.208
	Less	64 (16.9)	40 (10.6)			24 (13.8)	7 (4.0)		
Alcohol consumption^b^	More/equal	901 (41.5)	897 (41.3)	1.63	0.211	441 (54.4)	296 (36.5)	0.28	0.619
	Less	201 (9.3)	173 (8.0)			46 (5.7)	27 (3.3)		
Eat healthier	Agree	496 (18.4)	420 (15.6)	10.71	0.001	221 (18.0)	129 (10.5)	3.76	0.052
	Disagree	843 (31.3)	932 (34.6)			502 (40.8)	377 (30.7)		
Feel more energetic	Agree	923 (34.3)	731 (27.2)	65.17	< 0.001	467 (38.1)	281 (22.9)	9.95	0.002
	Disagree	412 (15.3)	623 (23.2)			255 (20.8)	223 (18.2)		
Chance of maintaining sport behaviour	Agree	949 (35.3)	868 (32.3)	13.30	< 0.001	538 (44.0)	339 (27.7)	7.33	0.007
	Disagree	389 (14.5)	481 (17.9)			183 (15.0)	163 (13.3)		
I know that performing sport is not my thing	Agree	21 (0.8)	28 (1.0)	0.97	0.387	12 (1.0)	14 (1.1)	1.82	0.226
	Disagree	1313 (49.1)	1316 (49.1)			711 (58.0)	488 (39.8)		
Feel better about myself	Agree	859 (32.0)	646 (24.1)	74.19	< 0.0001	492 (40.1)	257 (21.0)	37.60	< 0.0001
	Disagree	475 (17.7)	703 (26.2)			229 (18.7)	248 (20.2)		
Feel more like an athlete	Agree	605 (22.5)	422 (15.7)	55.40	< 0.0001	343 (28.0)	168 (13.7)	24.68	< 0.0001
	Disagree	731 (27.2)	926 (34.5)			377 (30.8)	335 (27.4)		
Changed lifestyle	Agree	913 (34.1)	796 (29.7)	25.01	< 0.0001	502 (40.9)	302 (24.6)	12.76	< 0.001
	Disagree	421 (15.7)	550 (20.5)			220 (17.9)	204 (16.6)		
Stimulating others to perform sport	Agree	657 (24.5)	566 (21.1)	14.65	< 0.001	384 (31.3)	217 (17.7)	12.02	0.001
	Disagree	676 (25.2)	784 (29.2)			339 (27.6)	287 (23.4)		
Losing weight	Agree	543 (20.2)	399 (14.8)	36.72	< 0.0001	270 (22.0)	125 (10.2)	21.61	< 0.0001
	Disagree	794 (29.5)	955 (35.5)			453 (36.9)	380 (30.9)		
Feel tired more often	Agree	97 (3.6)	84 (3.1)	1.17	0.282	52 (4.3)	38 (3.1)	0.08	0.824
	Disagree	1237 (46.1)	1266 (47.2)			668 (54.7)	463 (37.9)		

^a^The participants who did not smoke were excluded

^b^The participants who did not drink alcohol were excluded

### Predictive ability of app use

Table 3 presents results of the logistic regression analyses for each distance, corrected for age, gender, BMI, kilometres per week before preparation and frequency of participation in this running event. Logistic regression analyses showed that for both 16 and 6.4 km runners, app use was positively related to RPA and feeling healthier. In addition, the app use was related to feeling better about themselves, feeling more like an athlete, motivating others to participate in running, and losing weight. Also, for the 16 km runners using apps was related to eating healthier, feeling more energetic and reporting a higher chance to maintain sport behaviour.

**Table 3:** Results of multivariate logistic regression with outcome measure RPA, perceived health and lifestyle

		App use		
	Distance	OR (95 % CI)^a^	P	R^2b^
RPA	16 km	1.43 (1.16–1.75)	0.001	0.41
	6.4 km	1.89 (1.34–2.65)	<0.001	0.38
Health	16 km	1.59 (1.33–1.90)	<0.0001	0.10
	6.4 km	1.33 (1.02–1.73)	0.038	0.10
Alcohol consumption	16 km	1.06 (0.83–1.35)	0.651	0.04
	6.4 km	1.57 (0.86–2.85)	0.143	0.03
Smoking behaviour	16 km	1.09 (0.71–1.69)	0.691	0.06
	6.4 km	2.06 (0.80–5.30)	0.134	0.05
Eat healthier	16 km	1.24 (1.03–1.48)	0.022	0.02
	6.4 km	1.24 (0.93–1.66)	0.150	0.04
Feel more energetic	16 km	1.68 (1.40–2.01)	<0.0001	0.08
	6.4 km	1.13 (0.99–1.70)	0.055	0.05
I know that performing sport is not my thing	16 km	0.92 (0.44–1.75)	0.701	0.02
	6.4 km	0.47 (0.19–1.03)	0.058	0.12
Chance of maintaining sport behaviour	16 km	1.24 (1.03–1.50)	0.021	0.02
	6.4 km	1.31 (0.98–1.74)	0.067	0.02
Feel better about myself	16 km	1.75 (1.47–2.09)	<0.0001	0.07
	6.4 km	1.84 (1.41–2.40)	<0.0001	0.07
Feel more like an athlete	16 km	1.69 (1.41–2.01)	<0.0001	0.05
	6.4 km	1.67 (1.28–2.18)	<0.001	0.06
Did not change lifestyle	16 km	0.70 (0.58–0.83)	<0.0001	0.02
	6.4 km	0.70 (0.53–0.92)	0.010	0.06
Motivated others to participate	16 km	1.43 (1.20–1.69)	<0.0001	0.02
	6.4 km	1.45 (1.12–1.87)	0.005	0.03
Lost weight	16 km	1.57 (1.31–1.89)	<0.0001	0.06
	6.4 km	1.72 (1.29–2.30)	<0.0001	0.09
Feel tired more often	16 km	1.03 (0.73–1.46)	0.877	0.04
	6.4 km	0.70 (0.44–1.12)	0.140	0.03

^a^Controlled for gender, age, BMI, training sessions per year and weekly training distance before training phase

^b^Nagelkerke R^2^ [39]

## Discussion

Our main finding was that app use was positively related to RPA, feeling healthier, changing lifestyle and self-image. Also, use of apps was positively related to stimulating others to become active. Moreover, app use in 16 km runners was positively related to feeling more energetic, eating healthier and maintaining the sport behaviour. The odds ratios ranged from 1.24 to 1.89. Additionally, for RPA the explained variance was 41 % and 38 % for 16 km and 6.4 km respectively. These findings are of high importance considering that for app users the weekly training volume prior to the preparation phase was lower than non-app users.

These results corroborate with the findings of other studies, in which app use seemed to have increased physical activity and a healthy lifestyle [11, 14–17]. In contrast to those studies, the focus in this study was on mobile app use only. It should be noted that we did not analyse the effect of app use, but we examined the use of mobile apps in relation to physical activity, perceived health and self-image. This relationship between app use and perceived health and self-image in the preparation of a running event has not been considered in previous studies. Analysing this relationship is relevant, since it provides insight in innovative and accessible ways to encourage physical activity and a healthier life.

Although most results were comparable for 16 and 6.4 km runners, a few differences were found. In 16 km runners, app use was related to eating healthier, feeling more energetic and a higher chance to maintain sport behaviour. The relationships between app use and these variables did not reach significance level in the 6.4 km runners. The “fun run” character of the 6.4 km may be a first explanation for the differences found. Compared to the 16 km run, participation in a 6.4 km run may not require a long preparation phase and lifestyle changes. In addition, we found that in the training phase most 16 km runners trained 10–20 km per week (37.3 %) and 20–30 km per week (27.3 %), whereas the largest part of 6.4 km runners trained 5–10 km per week (42.0 %) and 10–20 km per week (26.3 %). Thus another possible explanation might be that the differences in weekly training distance of 16 and 6.4 km runners combined with a shorter preparation resulted in the inconsistent findings. Previous literature has shown that running improves aerobic fitness and cardiovascular function at rest [26]. In a review, a fairly strong dose–response relationship between weekly training volume and cardiorespiratory fitness was shown for inactive and healthy middle aged and elderly people [27]. This may explain why the physical fitness of the 16 km runners increased more compared to 6.4 km runners, resulting in a higher perceived energy level. Potentially, there is a link between weekly training volume and eating behaviour as well. To support this suggestion, Williams et al. showed that a larger weekly running distance promoted a healthier eating pattern [28]. In addition, in that study a relationship was found between weekly running distance and years spend in running, which might provide an explanation for our finding that app use was related to a higher chance to maintain the physical activity of the longer distance runners compared to the shorter distance runners.

Previous studies have shown that participating in running events can encourage physical activity [26, 29]. However, maintaining an active lifestyle is difficult for many [30]. Moreover, the gap between intention for being physically active and actually being active is large [31]. In many of behaviour change models, such as the Fogg behaviour model and the attitude, social influence and efficacy (ASE) model, the behavioural intention is assumed to be most important in changing behaviour [32, 33]. It would be interesting to determine the impact of an app on behaviour determinants such as self-efficacy, attitude and social influence. In addition, given that behaviour change theories (BCTs) are often relatively absent in apps, it would be valuable to find out which of these theories are taken into account in the app [34].

This study showed that the intention to maintain the running behaviour was higher for the app users, therefore app use may assist in decreasing drop-out of running and encouraging physical activity. This is a very interesting finding, since apps were more often used by overweight participants and the participants in the 6.4 km run (who trained less often). For these two groups physical activity may need to be encouraged. Furthermore, a very interesting finding was that app users more often encouraged others to engage in running compared to non-app users. This could be explained by the fact that some apps contain features to interact with others, such as following and supporting their activities [13]. This interaction combined with the use of social media might motivate others to be more active [35]. These findings suggest that the use of mobile apps can contribute to the promotion of running and prevention of drop-out. Our findings may be related to the new phenomenon of quantified self, which means that people are measuring their health conditions via wearables [36]. This new trend may actually be an underlying element in the findings of this study.

Furthermore, when we look at practical implications, we suggest that app use could be an additional stimulus to the training program, because it provides an easy and accessible tool to promote physical activity and a healthy lifestyle. Given that the use of smart phones increases [37, 38], a large amount of individuals can be reached with health and fitness apps. Sport organizations and employers may therefore recommend the use of apps in the preparation of a running event. For instance, large recreational running events often include a business run, in which business teams can compete. The use of apps may encourage employers to train more and live healthier. This data shows that app use is related to increased physical activity and improved health. Moreover, frequency of app use is higher in inexperienced and overweight participants. We could hypothesize that these group of runners have some comparable characteristics as inactive individuals. Therefore, our results could potentially be transferred to inactive individuals.

Some limitations of this study need to be addressed. At first, a self-reported, non-validated survey was used. Second, a causality between app use and the outcome variables cannot be determined. It remains unclear what would be the cause and what would be the result; did app use increase physical activity or did physical activity encourage app use. The involvement of other underlying causes should be considered as well. Randomized controlled studies need to be performed to determine a causal relationship. The third limitation was that several types of apps were included. The most used app was Runkeeper, but also a number of other apps were used. It would be interesting to find out why people choose certain apps and which features make an app popular. Apps differ in their features and may differ in their effectiveness as well. Therefore, the possibility that the relationships found might be different for each app has to be kept in mind, because the way apps present information and provide feedback differs. As a fourth limitation low explained variances for app use in relation to most of the health and lifestyle outcomes were found. Therefore we have to keep in mind that other factors, such as psychological factors, contributed to the runner’s lifestyle and self-image as well. At last, this study included individuals that were already active and motivated to participate to a running event. However, considering the problem of increased inactivity, it would be even more interesting to conduct research on potential of app use in promoting a healthy lifestyle in inactive individuals including long-term consequences. Further research is needed to determine which features would need to be included in such an app.

## Conclusion

In conclusion, our results showed that recreational runners who used an app are more likely to be more physically active and feel and live healthier. These results suggest that use of mobile apps has a beneficial role in the preparation of a running event, as it promotes health. Further research is now needed to determine a causal relationship between app use and physical and health related outcomes. More specific, a randomized controlled trial (RCT) needs to be developed and conducted. For instance, the effect of one app such as Runkeeper could be examined on weekly training distance and lifestyle. Another example would be to develop and evaluate a physical activity and health promotion app in a group of inactive individuals. To gain insight in long-term effects, a follow-up survey should be included as well.

## Additional files

Additional file 1:
**Online Survey Dam tot Damloop.** This file contains the questions that were stated in the online survey used in this study. The file starts with an introduction in which the purpose is explained and confidentiality is guaranteed. (PDF 239 kb)
