# Supplementary material for: App use, physical activity and healthy lifestyle: a cross sectional study
Source: BMC Public Health. 2015 Aug 28;15:833. doi: 10.1186/s12889-015-2165-8 (PMC4552464; doi:10.1186/s12889-015-2165-8)
Supplement: Additional file 1: — Online Survey Dam tot Damloop. This file contains the questions that were stated in the online survey used in this study. The file starts with an introduction in which the purpose is explained and confidentiality is guaranteed. (PDF 239 kb) [file 12889_2015_2165_MOESM1_ESM.pdf]

Additional file 1: Online Survey Dam tot Damloop

File format: pdf

Online Survey Dam tot Damloop 2014

The aim of this study is to gain insight in the positive and negative effects of training for the Dam tot Damloop on participants.

This study is conducted by the Amsterdam University of Applied Sciences. You are invited to participate in this study, because you are registered for the Dam tot Damloop 2014.

Your participation to this study is voluntarily. You can decide if you want to participate and you are allowed to quit at any time.

For this study, we ask you to fill in an online survey, this will take approximately 15 minutes. After 6 months we will send you another survey. By then, you can decide if you want to participate in this follow-up survey.

Your answers will be kept strictly confidential.

If you have questions about this study, please contact Dr. Marije Baart de la Faille – Deutekom (m.baart.de.la.faille@hva.nl).

Please indicate if you would like to participate in this research. If you do not want to participate, click on "no". If you mark "yes" this means you:

- have read the information described above
- participate voluntarily
- are 18 years or older

- Yes
- No

For which distance did you subscribe?

- 16 KM (10 EM)
- 6.4 KM (4 EM)
- Other (please give further information) …

How often did you participate in the Dam tot Damloop?

- This was the first time
- This was the second time
- This was the third time
- This was the fourth time
- This was the fifth time
- This was the sixth time or more often
- Do not know / no answer

Did you actually participate in the Dam tot Damloop?

- Yes
- No

What was the reason for not starting?

- Being sick
- Injury
- Overtraining
- I did not want to start
- Weather conditions
- Family or personal circumstances
- Other, namely …

Did you train for the Dam tot Damloop?

- Yes
- No

Did you finish the Dam tot Damloop?

- Yes
- No

What was your time at the Dam tot Damloop?
… hours
… minutes

Do you usually take your phone with you during running?

- Yes
- No

Did you use an app for exercising?

- Yes
- No

Which app did you use during training for the Dam tot Damloop?

- Dam tot Damloop 2014 app
- Myasics
- Adidas miCoach
- RunKeeper
- Get Runningapp
- Nike + iPod / I Phone app
- Runtastic
- Strava
- Endomundo
- App with Renate Wennemars: Running Coach powered by the athletics union

Could you indicate what the duration of the period of training was for the Dam tot Damloop?

- Did not train or barely
- Trained 1-5 weeks
- Trained 6-11 weeks
- Trained 12 weeks or more
- No separate training period (I exercise throughout the whole year)
- Do not know / no answer

We are interested in the consequence of your participation in the Dam tot Damloop on amount of physical exercise.

How much kilometres did you train per week prior to your training period for the Dam tot Damloop?	Less than 5 km per week	5-10 km per week	10-20 km per week	20-30 km per week	More than 30 km per week	Do not know / no answer
How much kilometres did you train per week during your training period for the Dam tot Damloop?	Less than 5 km per week	5-10 km per week	10-20 km per week	20-30 km per week	More than 30 km per week	Do not know / no answer

Do you think that training for the Dam tot Damloop had an effect your health?

- No effect
- Yes, I feel much healthier
- Yes, I feel healthier
- Yes, I feel less healthy
- Yes, I feel much less healthy

In total, how many times did you perform sports during the last 12 months?
If you do not know the exact number, please give an estimation that is as accurate as possible.

… times

We are interested in the effect of your participation in the Dam tot Damloop on the behaviour that influences your health.
Previously, questions about sports and exercise have been asked. That is why we, in the next section, ask for other aspects of behaviour that may have been influenced by the Dam tot Damloop.

Alcohol consumption

On average, how many glasses of alcohol did you drink per week prior to your training period for the Dam tot Damloop?	None	1-3 glasses per week	4-7 glasses per week	8-14 glasses per week	More than 14 glasses per week	Do not know / no answer
On average, how many glasses of alcohol did you drink per week during your training period for the Dam tot Damloop?	None	1-3 glasses per week	4-7 glasses per week	8-14 glasses per week	More than 14 glasses per week	Do not know / no answer

Smoking behaviour

How often did you smoke prior to your training period for the Dam tot Damloop?	Never	Occasionally	1-3 pieces a day	4-10 pieces a day	More than 10 pieces a day	Do not know / no answer

How often did you smoke during your training period for the Dam tot Damloop?	Never	Occasionally	1-3 pieces a day	4-10 pieces a day	More than 10 pieces a day	Do not know / no answer

To what extent do you agree with the following theses in relation to the training for the Dam tot Damloop?

I eat healthier.	Totally agree	Agree	Neutral	Do not agree	Totally do not agree
I feel more energetic.	Totally agree	Agree	Neutral	Do not agree	Totally do not agree
I know that performing sports is not my thing.	Totally agree	Agree	Neutral	Do not agree	Totally do not agree
The chance is high that I will keep on performing sports on the long-term.	Totally agree	Agree	Neutral	Do not agree	Totally do not agree
I feel better about myself.	Totally agree	Agree	Neutral	Do not agree	Totally do not agree
I see myself more as an athlete.	Totally agree	Agree	Neutral	Do not agree	Totally do not agree
I did not change anything in my lifestyle.	Totally agree	Agree	Neutral	Do not agree	Totally do not agree
I encouraged others in my surrounding to perform sports.	Totally agree	Agree	Neutral	Do not agree	Totally do not agree
I lost weight.	Totally agree	Agree	Neutral	Do not agree	Totally do not agree
I feel tired more often.	Totally agree	Agree	Neutral	Do not agree	Totally do not agree

Are you a man or a woman?

- Man
- Woman

Wat Is your body height in centimetres at this moment?

… cm

What is your body weight in kilogrammes at this moment?

… kg

What is your year of birth?

….
